# Spontaneous Superficial Femoral Artery Mycotic Aneurysm

**DOI:** 10.1155/2021/6613914

**Published:** 2021-02-25

**Authors:** Kalpana Vijaykumar, Jia Sheng Tay, Tjun Yip Tang, Edward Tieng Chek Choke

**Affiliations:** ^1^Vascular and Endovascular Service, Department of Surgery, Sengkang General Hospital, Singapore; ^2^Vascular Surgery, Division of Surgery & Surgical Oncology, Singapore General Hospital, Singapore

## Abstract

Peripheral artery mycotic aneurysms are rare occurrences. In this case, we review a 52-year-old lady with poorly controlled diabetes who developed a spontaneous left superficial artery mycotic aneurysm. She underwent excision and subsequent extra-anatomic bypass with a great saphenous vein graft. She had full functional recovery after a short period of rehabilitation.

## 1. Introduction

The term mycotic aneurysm, first coined by Osler in 1885, refers to localized, irreversible arterial dilatation due to destruction of the vessel wall by infection [1]. This may occur in a previously healthy artery or may be due to secondary infection of a preexisting aneurysm. Various risk factors including direct arterial injury, antecedent infection, preexisting conditions resulting in impaired immunity, or atherosclerotic disease predispose patients to mycotic aneurysms [2].

In this case, we reviewed a 52-year-old lady with poorly controlled diabetes who developed a spontaneous left superficial femoral artery (SFA) mycotic aneurysm.

## 2. Case Report

A 52-year-old lady with hypertension, diabetes, and asthma presented with a one-week history of left groin pain. The pain started insidiously and was localized to the left groin. She was otherwise well and asymptomatic prior to this. There was no history of preceding trauma. Of note, she had a dental extraction procedure two weeks prior to this. She was only on inhaled corticosteroids for her asthma, without any systemic corticosteroids.

On examination, she was found to have a tender left upper thigh mass that measured about 4 × 4 cm. Other than this, her lower limb neurovascular examination did not reveal any abnormalities. She did not have any features of distal emboli or splinter hemorrhages.

Initial blood investigations revealed a total white cell count of 18.6 × 10^9^/L as well as a raised C-reactive protein of 247.7 mg/L. Her diabetic control was also poor with a glycosylated hemoglobin level of 15.4%. Initial blood cultures were also taken which did not reveal any microbes.

**Figure 1:**
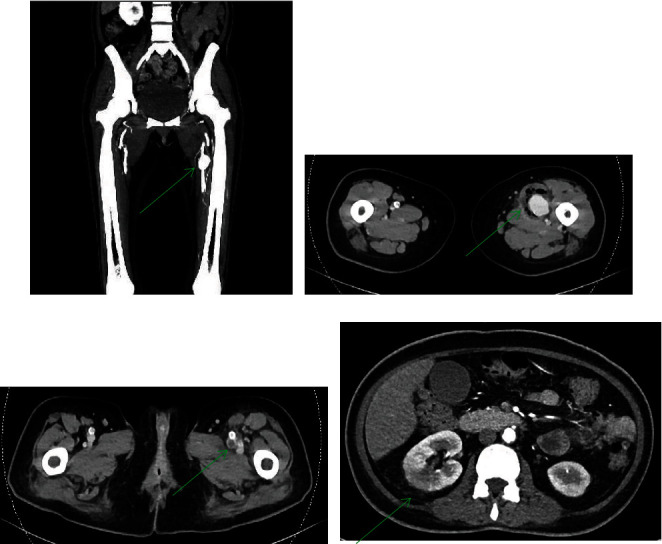
(a) Arrow indicates saccular pseudoaneurysm of superficial femoral artery. (b) Arrow indicates gas containing intramuscular fluid collection surrounding the pseudoaneurysm. (c) Arrow indicates filling defect within the common femoral vein. (d) Arrow indicates hypoenhancing area in the right kidney with surrounding fat stranding.

She underwent an urgent computed tomographic (CT) angiogram of the lower limbs which revealed a thin wall eccentric saccular pseudoaneurysm arising from the proximal third of the left SFA measuring 2.0 × 2.6 × 3.0 cm with a gas containing intramuscular fluid collection seen. Mass effect from the collection and pseudoaneurysm was compressing the adjacent femoral vein causing central intraluminal filling defects, suspicious of deep venous thrombosis (Figure 1).

A CT aortogram was done to rule out any other mycotic aneurysms. This revealed moderated intimal calcification of the infrarenal abdominal aorta and bilateral common iliac arteries. In addition to this, a wedge-shaped hypoenhancing area in the right renal inferior pole with adjacent fat stranding was noted, suggestive of possible pyelonephritis. An echocardiogram was also done, which did not reveal any vegetations.

Urinalysis was performed but no microbes were detected. She was started on intravenous augmentin after the blood cultures were taken.

**Figure 2:**
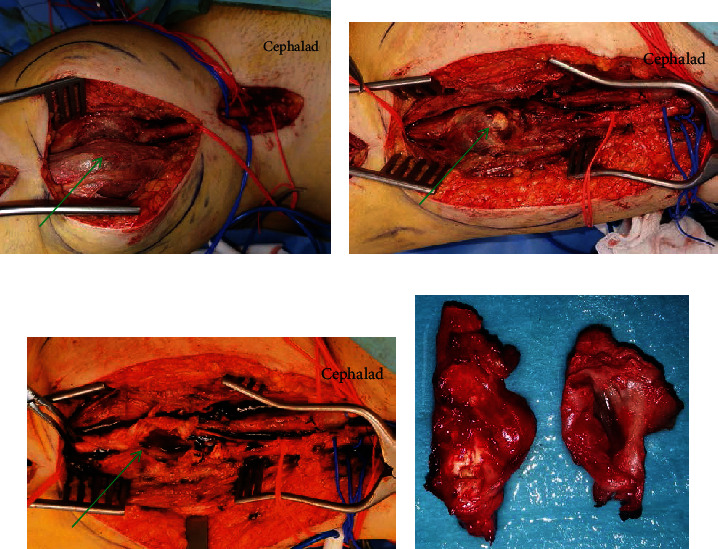
(a) Arrow indicates intramuscular pseudoaneurysm. (b) Arrow indicates ulcerated portion of pseudoaneurysm. (c) Arrow indicating turbid fluid from mycotic pseudoaneurysm. (d) Excised pseudoaneurysm which was confirmed as a mycotic pseudoaneurysm on histopathological examination.

She was planned for and underwent excision of the left SFA mycotic pseudoaneurysm with above knee femoropopliteal bypass with a superficial femoral vein thrombectomy. We made 2 separate incisions—a longitudinal groin incision to obtain proximal control and a longitudinal above knee incision to obtain distal control and access the aneurysm. Intraoperatively, she was found to have a 3 × 3 cm intramuscular pseudoaneurysm with a surrounding abscess cavity containing pus (Figure 2). During mobilization, the aneurysm had ruptured; hence, it was dealt with at that point in time instead of doing a bypass first. A short segment of the superficial femoral vein was noted to be thrombosed intraoperatively, and thus a venotomy and thrombectomy were done for this. This was not detected preoperatively, and hence no deep venous scans were performed ahead of time.

**Figure 3:**
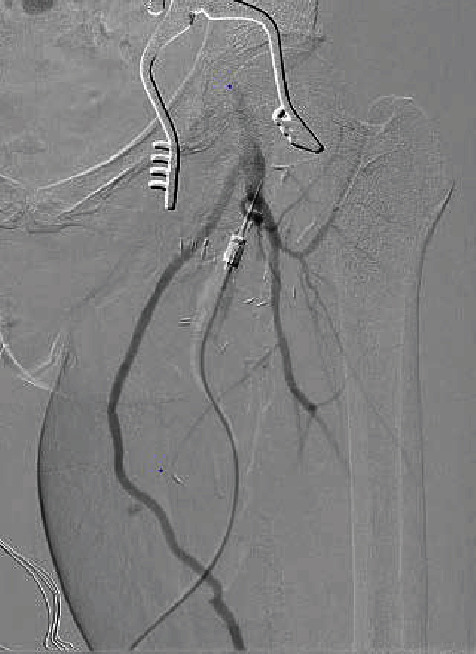
Diagnostic angiogram showing extra-anatomic bypass.

Extra-anatomic bypass with the great saphenous vein was to avoid the infected field and allow for partial closure of the wound without exposing the graft (Figure 3).

Intraoperative blood cultures were taken from the pseudoaneurysm yielded pan-sensitive Escherichia Coli. Tissue cultures of the aneurysm also revealed the same organism.

**Figure 4:**
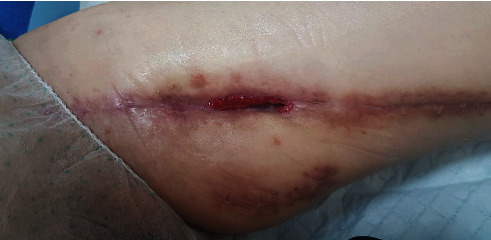
Postoperative wounds 10 weeks after surgery.

Postoperatively, she was monitored in the high dependency ward for a few days and then subsequently transferred to the general ward. Antibiotics were tailored based on the cultures and she completed a six-week course of intravenous cefazolin. Regular dressing change was performed for her wound initially, and this was then converted to negative pressure wound therapy. With this, her wound was healing well (Figure 4). Functionally, she was able to ambulate after a short period of rehabilitation.

## 3. Discussion

Mycotic pseudoaneurysms of the peripheral arteries are rare and can occur as a result of localized infection or systemic sepsis [3]. With the advent of antibiotics, there are fewer pseudoaneurysms as a result of infective endocarditis and more resulting from direct arterial injury [ 2] .However, the occurrence of pseudoaneurysms in native vessels in a patient without antecedent direct arterial injury is still very rare, as in our case.

In a study of 57 patients with infected pseudoaneurysms [5], pain was the predominant symptom (83.3%). This was followed by oedema (80%) and erythema (78.5%) with only 30% of patients having fever. This was similar to what our patient presented with.

In a series of 26 patients by Brossier [6], blood cultures were reported positive in only 61% of patients; however, organisms were isolated from the aneurysm and surrounding tissue in 84% of patients. While bacteriological patterns continue to evolve, organisms such as Staphylococcus (28%) and Salmonella (15%) remain the most common [2]. In a review of the literature thus far, very few case reports mention Escherichia Coli (E.coli) being a cause of mycotic aneurysms. In a case report by McCann [7], they reviewed two patients with multidrug-resistant E.coli, one of whom had intrathoracic pseudoaneurysms while the other had a right common carotid pseudoaneurysm following prolonged E Coli septicemia. Our patient, however, did not present with E Coli septicemia and was well prior to developing a SFA pseudoaneurysm, which was rather unusual. In our patient, she had prior dental extraction and evidence of pyelonephritis on the CT scan. However, neither her blood nor urine cultures revealed any significant bacterial growth.

The aim of surgical management of infected pseudoaneurysms is similar to management of infected vascular grafts, such that all infected or necrotic tissue should be adequately debrided and resultant ischemia dealt with. In the case of our patient, given that she did not have existing peripheral vascular disease, ligation without revascularization would have resulted in a debilitating state for her. In an American study by Benjamin et al. [8], superficial femoral and proximal popliteal veins were used as a conduit for limb vascularization in patients with mycotic pseudoaneurysms with good outcome. Usually, the contralateral great saphenous vein is chosen as a conduit [9, 10], resulting in bilateral incisions and wounds. In our patient, we decided to proceed with the ipsilateral great saphenous vein with good outcome. Hence, it is an option to be considered when performing extra-anatomic bypass with an autologous graft for a patient with mycotic pseudoaneurysms.
